# Celiac disease in autism spectrum disorder: data from an Italian child cohort

**DOI:** 10.1186/s13052-023-01484-x

**Published:** 2023-07-03

**Authors:** Stefania Zambrano, Barbara Parma, Valeria Morabito, Silvia Borini, Roberta Romaniello, Massimo Molteni, Elisa Mani, Angelo Selicorni

**Affiliations:** 1 Scientific Institute, IRCCS E. Medea Child Psychopathology Unit, Bosisio Parini, Lecco, Italy; 2 Postgraduate Specialization School in Child and Adolescent Neuropsychiatry, Università degli Studi di Milano, Milano, Italy; 3 Department of Pediatric, Mariani Foundation Center for Fragile Child ASST-Lariana, Sant’Anna Hospital, San Fermo della Battaglia, Como, Italy; 4 Child Neuropsychiatry Unit, Department of Mental Health, ASST-Lariana, Sant’Anna Hospital, San Fermo della Battaglia, Como, Italy

**Keywords:** Autism, Neurodevelopmental disorder, Children, Gut-brain axis, Celiac disease, Gluten, Autoimmunity, TGA, TTG, EMA

## Abstract

**Background:**

In recent decades some studies described the frequent co-occurrence of celiac disease autoimmunity and overt celiac disease in patients with autism. Therefore, it was suggested that celiac disease could play a possible role in the etiopathogenesis of autism spectrum disorder. However, several other studies have not confirmed this association. The aim of the present study was to elucidate the potential association between autism spectrum disorder and celiac disease.

**Methods:**

We prospectively collected data from an Italian cohort of 223 children at the time of their clinical diagnosis of autism spectrum disorder in the 2019–2020 period. A serological celiac disease screening was performed and data were available for 196 patients; male (M):female (F) ratio = 4.4:1; median age = 3.6 years; age range = 1.6–12.8 years. Full-blown celiac disease was established according to the diagnostic algorithm of the European Society of Paediatric Gastroenterology, Hepatology and Nutrition (ESPGHAN) 2012 or 2019 guidelines. Fisher’s exact test was used to compare the celiac disease seroprevalence and prevalence in our autism spectrum disorder cohort and in the Italian healthy pediatric population studied by Gatti et al. to highlight the possible differences between the two groups.

**Results:**

A not statistically significant difference between the celiac disease seroprevalence in our autism spectrum disorder cohort (4.08%) and Gatti’s Italian healthy group (2.22%) was found, p = 0.0810; OR = 1.871. A similar result emerged for overt celiac disease prevalences (2.24% versus 1.58%, respectively), p = 0.2862; OR = 1.431.

**Conclusions:**

Our data validates a weakness of association between autism spectrum disorder and celiac disease. On the basis of our results, a regular screening for CD in patients with ASD is not recommended to a greater extent than in the general population.

**Supplementary Information:**

The online version contains supplementary material available at 10.1186/s13052-023-01484-x.

## Background

Autism Spectrum Disorder (ASD) is among the most widespread neurodevelopmental disorders, with an overall prevalence estimated to be 18.5 per 1000 (1 in 54) children aged 8 years, varying from 13.1 to 31.4 per 1000 children among the Centers for Disease Control and Prevention (CDC)-established Autism and Developmental Disabilities Monitoring (ADDM) network sites [1] and an average male:female ratio of 4:1 [2]. According to an Italian study, ASD prevalence in Italy is 1 in 87 children aged 7–9 years [3]. Symptoms typically manifest in the early developmental period. According to The Diagnostic and Statistical Manual of Mental Disorders (DSM-5), they include persistent difficulties in social engagement and social interaction and restricted, repetitive patterns of behaviors and activities [4, 5]. As evidence suggests, a number of environmental and genetic factors may potentially contribute to the etiopathogenesis of ASD [2, 4, 6]. Comorbidities with other neuropsychiatric disorders are frequently observed, including Attention-Deficit/Hyperactivity Disorder (ADHD), intellectual disability, mood and/or behavior disorders, motor abnormalities, epilepsy and sleep disorders [7]. Moreover, co-occurrence of ASD and celiac disease (CD) is described in several studies [8–10].

CD is a chronic autoimmune disorder caused by gluten intolerance in subjects with genetic susceptibility linked to the Human Leukocyte Antigen (HLA)-DQ2 and/or DQ8 haplotypes [11]. It is spread all over the world with different rates in various countries and is estimated at 0.14% up to 5.7% with an average female (F):male (M) ratio of 2.8:1 [12, 13]. Overall, CD prevalence is around 1% (95% Confidence Interval (CI): 0.9–1.1) of the total European population and 0.9% (95% CI: 0.6–1.2) of the European child population [14]. Mustalahti et al. calculated a CD prevalence of about 1.1% (95% CI: 0.7–1.5) among Italian children aged 0–16 years [14]. About ten years later, Gatti et al. found a CD prevalence of 1.58% (95% CI: 1.26-1.90%) in an Italian mass screening study with a cohort of 5,705 children aged 5.0–11.0 years [15]. The causes of this large increase in CD incidence over the past 25 years have not yet been clarified [15].

CD primarily affects the small intestine leading to a chronic inflammatory enteropathy with clinical symptoms and signs of malabsorption, such as weight loss, chronic diarrhea, bloating, abdominal distension or pain, known as “typical manifestations”. But it may affect all physiological systems, with signs known as “atypical manifestations” (e.g. constipation, infertility, dermatitis herpetiformis) [16].

In CD, dysfunction of the intestinal barrier owing to defective tight-junctions seems to be responsible for an increased intestinal permeability (“leaky gut”), which allows the gliadin to reach the lamina propria and activate an inflammatory cascade promoting the synthesis of autoantibodies (e.g. Anti-Transglutaminase Antibodies (TGA), Anti-Gliadin (AGA), Anti-Endomysium (EMA)) [13]. This condition causes damage to a number of different organs and primarily the gut [16]. Moreover, since people with CD present an imbalance in the intestinal bacterial flora (“dysbiosis”), with a reduction of Bacteroides spp. and Bifidobacteria spp., some authors hypothesized that this may play a role as a co-factor in the inflammatory process underlying the disorder [17]. This led to the theory of the “brain-gut-microbiota axis” that sustains a bidirectional interaction between brain and gut-microbiota [18]. Changes in the intestinal flora composition and metabolites may be responsible for atypical pro-inflammatory immune signalling that may alter the functioning of the blood-brain barrier and of immune and neural cells in the Central Nervous System (CNS), resulting in an anomalous CNS development and in neurological and/or psychiatric disorders [19]. Furthermore, it is known that CD is clinically associated with neuropsychiatric disorders such as cerebellar ataxia, myopathy, peripheral neuropathy, cognitive impairment, headache, seizures and symptoms such as hyperactivity, irritability and aggressive behavior [20, 21]. On the other hand, clinical experience shows that subjects with ASD often report gastrointestinal symptoms such as constipation, flatulence, diarrhea, incontinence, abdominal pains (22.7–70.0% of estimated prevalence) [22]. Regarding these aspects, it must be noted that patients with communication and/or speech difficulties and/or intellectual disability may present irritability not only as a clinical manifestation of their neuro-psychiatric disorder, but also as a symptom of physical pain [23].

Given the above, several papers were published over the past decades suggesting a possible link between ASD and CD and a supposed increased prevalence of CD in ASD. However, previous medical literature is far from clear. Ludvigsson et al. did not find any association between previous ASD and overt CD (OR: 0.93; 95% CI: 0.51–1.68), but identified a link with a positive CD serologic screening in subjects with normal gut mucosa (OR: 4.57; 95% CI: 1.58–13.22) [24]. Moreover, according to their data, an isolated CD serological autoimmunity was predictive of an increased risk of later ASD (Hazard Ratio (HR): 3.09; 95% CI: 1.99–4.80), in particular within the first year of age (HR: 7.05; 95% CI: 1.31–37.87) [24]. Batista et al. found no co-occurrence of ASD-CD in subjects with CD or autism compared with the USA general population (ASD prevalence among CD individuals was 0.95% vs. the USA population 0.90%; CD prevalence among ASD individuals was 0.00% vs. the USA population 0.54%) [25]. Similar results were obtained by Pavone et al. [26]. A recent retrospective cross-sectional study conducted in a large Italian cohort of school-aged children with ASD (mean age 7.2 years) ruled out a significant difference in the prevalence of CD compared to the Italian general population (2.18% vs. 1.58 respectively, P-value (P) = 0.36) [27]. On the contrary, Barcia et al. and Calderoni et al. found a statistically significant difference between overt CD prevalence in ASD individuals and healthy children, with outcomes of 3.3% (P = 0.014) and 2.26% (P = 0.0246) respectively [8, 10].

The aim of our study was to detect the full-blown CD prevalence and the CD seroprevalence in a large cohort of Italian children with ASD and compare our results with those of the Italian healthy pediatric population [15] to clarify the potential association between ASD and CD.

## Methods

### CD autoantibodies, gut biopsy and HLA-typing: technical details

Serological screening for CD consisted in the TGA-reflex test, which means that subjects with normal plasmatic levels of immunoglobulin (Ig)A were automatically screened for TGA-IgA levels. If the latter were above the upper limit of normal, EMA-IgA test was performed automatically. In patients aged less than two years, the AGA-IgA and IgG were dosed if the TGA-IgA-reflex test was negative. The EMA assay was carried out by Indirect Immunofluorescence Technique (IFI) on cryostatic sections of distal III of monkey esophagus and we considered “-“ as a negative result, “+” as a positive result (the plasmatic titer was recorded, when available). Gut biopsy was performed on at least 4 biopsies from the distal duodenum and at least one from the bulb obtained by Esophagus-Gastro-DuodenoScopy (EGDS) under sedation [28, 29]. The small-bowel mucosal lesions typical for CD were classified according to the Modified Marsh Classification by Oberhuber et al. [30]. Molecular typing of the HLA to detect CD-specific HLA-DQA1 and HLA-DQB1 haplotypes was performed by Polymerase Chain Reaction to Sequence Specific Primers (PCR-SSP).

### Participants, Inclusion/ exclusion criteria and study design

We conducted a longitudinal case-control study. Patients’ parents were asked for consent to use of the data for research purposes in anonymous and aggregated form. Data were prospectively collected from 01 to 2019 to 12 Mar 2020. We enrolled children who received a clinical diagnosis of ASD at the Child Psychopathology Unit of Scientific Institute, IRCCS Eugenio Medea (Bosisio Parini, Italy). ASD diagnosis was established with a clinical assessment by an experienced multidisciplinary team according to DSM-5 criteria [5] and confirmed by Autism Diagnostic Interview-Revised (ADI-R) and/or Autism Diagnostic Observation Schedule-Second Edition (ADOS-2).

On admission, a serological screening for CD was performed. We excluded patients who were receiving a Gluten-Free Diet (GFD) and subjects with syndromes/illness with known risk for CD [31].

For eligible subjects, we collected data about sex, age, family history of CD and diet; besides, we evaluated the presence of typical and atypical CD manifestations. All patients with a positive CD serological screening underwent a pediatric gastroenterologist examination and follow-up in the Department of Pediatrics - ASST-Lariana, Sant’Anna Hospital, San Fermo della Battaglia (Como). To confirm/exclude a diagnosis of CD, we used the diagnostic algorithm of the European Society of Paediatric Gastroenterology, Hepatology and Nutrition (ESPGHAN) 2012 or the ESPGHAN 2019 guidelines, depending on whether the subjects were enrolled before or after the release of the latest guidelines [29, 32]. Finally, we calculated the seroprevalence and the prevalence of CD in our cohort of children with ASD and compared our results with those obtained by Gatti et al. at the mass screening of healthy Italian children [15], to highlight a potentially higher CD seroprevalence and/or prevalence in the group of ASD children. For the same scope, we finally calculated the two Odds Ratios (OR). The eligible cohort by Gatti et al. was composed of 5,705 children aged 5.0–11.0 years. In agreement with the 2012 ESPGHAN guidelines, the authors found a CD seroprevalence of 2.22% (95% CI: 1.79–2.65%) in the screened patients’ group (4316 subjects) and a full-blown CD prevalence of 1.58% (95% CI: 1.26-1.90%) in the eligible sample [15].

### Statistics

We performed Fisher’s exact test-one-sided P-value to compare the seroprevalence and the prevalence of CD in our cohort and in the Italian sample healthy children by Gatti et al. Probability (P-value) less than 5% (< 0.05) was used to define a statistically significant difference between groups.

All data were analyzed with GraphPad Prism 8 (for Windows 64-bit – Version 8.4.1 (676), March 13, 2020 – ©GraphPad Software, 2365 Northside Dr. Suite #560, San Diego, CA 92,108, United States).

## Results

The eligible sample was composed of 223 patients, 181 males (81.2%) and 42 females (18.8%). Screening data for CD were available in 196 patients, 160 males (81.6%), 36 females (18.4%), defining an average M:F ratio of 4.4:1, with a median age of 3.6 years (mean age 4.3 years; mode: 4.0 years; standard deviation: 2.3 years; range: 1.6–12.8 years).

CD seroprevalence was established in 8 out of 196 screened subjects (cases), equal to 4.08% (CI 95%: 1.95 to 7.98). All patients were males. A diagnosis of full-blown CD was confirmed in 5 of them; 1 patient refused the gut-biopsy. Since he had a positive autoimmunity (both anti-TGA and anti-EMA, with decreasing antibodies titers at subsequent follow-ups), he was counted only among subjects with a positive CD serological screening. Thus, the overt CD diagnosis was definitely established in 5 out of 223 patients (cases), equal to 2.24% (CI 95%: 0.81 to 5.29). In Table 1., we report the data of all patients with a positive CD biochemical screening associated to their main features (age at CD screening, autoantibodies titers, symptoms/signs, gut biopsy data, HLA data, confirmed or excluded CD).

**Table 1:** ASD patients with a positive CD biochemical screening, with/without CD diagnosis: details

No.	Age (years)	TGA IgA (UI/mL)	EMA (titre)	CD Symptoms-Signs	Gut Byopsy	HLA DQ2/DQ8 Haplotypes	Familiarity for CD	CD Diagnosis
**1**	2.84	71.7	01:10	mild sideropenic anemia, low vitamin D	n.a.	n.a.	**-**	YES*
**2**	6.35	56.9 **-**	**-** **-**	constipation, dubious episodes of abdominal discomfort	n.p.	**+**	**-**	NO
**3**	4.93	127.6	**+** (moderate positive)	loose stool	n.p.	n.p.	**-**	YES*
**4**	2.82	200.0	01:24	**-**	+ (Marsh 3c)	n.p.	**-**	YES
**5**	2.54	24.5 **-**	01:10 **-**	**-**	n.p.	+	**-**	NO
**6**	3.02	75 54.9 46.2	01:20 01:10 01:10	**-**	refused	+	**-**	n.a.
**7**	5.01	200.0	01:320	constipation	n.p.	n.p.	**+**	YES
**8**	5.05	24.8 180.0	01:10 01:40	**-**	**+** (Marsh 3b)	n.p.	**-**	YES

ASD = autism spectrum disorder; CD = celiac disease; No.=number/Id of patient; TGA = anti-Transglutaminase Antibodies; EMA = anti-Endomysium antibodies; HLA = Human Leukocyte Antigen. Cut-off values for TGA: positive > 10 UI/mL; borderline 7–10 UI/mL; negative < 7 UI/mL; “+"= positive result/yes, “-“= negative result/none; “n.p.“= not performed, “n.a.“= not available, “*"= patient who had received a CD diagnosis in other Institute and lacking some data.

Counting the subjects screened less seropositive individuals (in total 188 patients), 5 patients had familiarity for CD, 35 patients had atypical and/or typical gastrointestinal symptoms corresponding to a global prevalence of 17.5% (in particular, 2 patients had both). Atypical CD symptoms were more common and were found in 24 subjects among all symptomatic patients (equal to 72.7%). The most common typical CD symptoms were abdominal floating (6 subjects), diarrhea (3 subjects) and poor weight gain (2 subjects). The most frequent atypical symptom was constipation (found in 17 patients), which was also the most common clinical manifestation.

Fisher’s exact test detected a not statistically significant difference (P > 0.05) between our CD seroprevalence in the ASD cohort and that of Gatti’s Italian healthy group (P-value: 0.0810 – Odds Ratio (OR): 1.871, CI 95%: 0.8953 to 3.749). A similar not significant result was seen when comparing our overt CD prevalence in ASD patients and that of Gatti’s Italian heathy subjects (P-value: 0.2862 – OR: 1.431, CI 95%: 0.6174 to 3.431) – see Table 2.

Among the 188 seronegative CD patients (controls), 5 patients had familiarity for CD, 33 patients had atypical and/or typical gastrointestinal symptoms corresponding to a global prevalence of 17.5%. Atypical CD symptoms were more common and were found in 24 subjects among 33 symptomatic patients (equal to 72.7%). The most frequent atypical symptom was constipation (found in 17/24 subjects; 70.8%), which was also the most common clinical manifestation. The most common typical CD symptoms were abdominal floating (6/9 subjects; 66.7%), diarrhea (3/9 subjects; 33.3%) and poor weight gain (2/9 subjects; 22.2%).

Fisher’s exact test detected a not statistically significant difference (P > 0.05) between our CD seroprevalence in the ASD cohort and that of Gatti’s Italian healthy group (P: 0.0810 – Odds Ratio (OR): 1.871, CI 95%: 0.8953 to 3.749) [15]. A similar not significant result was seen when comparing our overt CD prevalence in ASD patients and that of Gatti’s Italian heathy subjects (P: 0.2862 – OR: 1.431, CI 95%: 0.6174 to 3.431) [15] – see Table 2.

**Table 2:** CD seroprevalence and prevalence: results and comparison between the two cohorts

	CD-Cases (#)	Controls (#)	Prevalence – mean (%)	CI 95% (%)	Range of age (years)	Fisher’s exact test
**ASD cohort (our study)**						
***SEROPREVALENCE*** ***PREVALENCE***	8 5	188°^1^ 218°^2^	**4.08** **2.24**	1.95 to 7.98 0.81 to 5.29	1.6–12.8 1.6–12.8	P-value* / **OR (CI 95%)**
**Healthy control group (Gatti et al.)**						
***SEROPREVALENCE*** ***PREVALENCE***	96 90	4220 5615	**2.22** **1.58**	1.79 to 2.65 1.26 to 1.90	5.0–11.0 5.0–11.0	0.0810* / **1.871 (0.8953 to 3.749)** 0.2862* / **1.431 (0.6174 to 3.431)**

CD = celiac disease; ASD = autism spectrum disorder; # =number of subjects; CI = Confidence Interval; OR = Odds Ratio;

°1= seronegative CD subjects among those screened; °2= ASD subjects without overt CD from the eligible ASD cohort;

* A Probability (P)-value less than 0.05 (≤ 0.05) is statistically significant

## Discussion

In recent decades, CD has been proposed to play a possible role in the etiopathogenesis of ASD. This hypothesis emerged after some authors observed a high occurrence of gastrointestinal symptoms in presence of an increased prevalence of overt CD, and/or of autoantibodies against gluten, and/or an altered intestinal barrier/gut-microbiota in the population with autism [10, 15, 22]. These findings fitted well with the XIX – XXI-century emerging theory of the “brain-gut-microbiota axis” [33] whereby dysbiosis may be responsible for an atypical pro-inflammatory mechanism causing an abnormal function of the blood-brain barrier and of the neural immune cells in the CNS, resulting in an anomalous CNS development and neurological and/or psychiatric disorders [18, 19]. Furthermore, some recent evidence shows that CD may lead to neuropsychiatric manifestation such as seizures, ataxia, irritability, hyperactivity, etc. [20, 21].

These findings were however not confirmed in several other studies [25–27]. The aforementioned debated theories, on the one hand, encouraged clinicians to largely screen ASD patients for CD autoimmunity and, on the other, promoted several research works with very controversial results [25, 26, 33, 34].

The aim of our study was to detect the CD seroprevalence and prevalence in young subjects with autism and verify the existence, or otherwise, of a relation between CD and autism spectrum disorder.

Regarding the past and current literature about CD in this set of patients, it is interesting to note that our cohort represents a large sample. We therefore carried out one of the very few mass ASD population study, in which data were collected prospectively and then compared with those of a mass screening in the Italian sample of healthy children (by Gatti et al.) [15].

Globally, we found a slightly lower percentage of ASD patients with gastrointestinal symptoms (19.9%) compared to Adams et al. (22.7-70%) [22]. We retain that the lower rate is due to the very young age of our patients and therefore to their difficulty in expressing their possible gastrointestinal symptoms. Anyway, considering the subset of ASD subjects with overt-CD, symptomatic patients were more common than asymptomatic cases (60.0% vs. 40.0%). Interestingly, atypical symptoms, and in particular constipation, prevailed both in the serologically positive/overt CD subsets and in the ASD controls, suggesting a possible etiology other than CD and/or CD autoimmunity [22].

Like most published papers [35], our results validate the lack of a real association between ASD and CD, both with regard to CD autoimmunity, and in terms of fully-blown CD. Specifically, we found a CD seroprevalence of 4.08% against the 2.22% in the Italian cohort of healthy children by Gatti’s et al., and a CD prevalence of 2.24% versus their 1.58% rate (P > 0.05 in both cases).

Looking at our data and those of other colleagues, we noticed some very important issues: first, ASD is more frequent in males than in females, but most research studies (like ours) compared the prevalence in ASD patients with the prevalence obtained from mass screenings for CD in healthy children, which were composed by similar rates of males and females; however, a female predominance in CD is widely recognized. This generates a selection bias. Secondly, the vast majority of studies in the literature are subject to other biases related to the low number and the mean young age of the patients enrolled. The large Confidence Intervals at 95% we obtained are primarily related to the relatively small number of subjects in our sample. Nevertheless, our ASD cohort is among the largest ever enrolled, and this provides the idea of the burden exerted by a singular case of overt CD on the calculation of the total CD prevalence, that we can summarize in a surveillance bias. The last relevant issue regards the mixed results achieved by different authors, that are partially related to changes in the diagnostic approach and guidelines for CD in the years, but especially to the different definitions of “serological CD”, “overt CD” and “doubtful cases” used by researchers. CD seroprevalence and/or CD prevalence rates are thus determined according to various methodologies depending on specific studies and can thus generate information biases.

In recent years, some studies supported the evidence of an increased immune reactivity and sensitivity against gluten in ASD subjects, even in the absence of full-blown CD. In their paper, de Magistris et al. found an increased prevalence of AGA-IgG and DPG-IgG in patients with autism spectrum disorder as compared to the healthy population [36]. A few years later, Józefczuk et al. demonstrated a lack of deterministic correlation between the presence of gluten-induced autoantibodies and an increased intestinal permeability in ASD patients, in opposition to the hypothesis of so-called “leaky gut” [37]. The authors maintained that, while intestinal Tissue TransGlutaminase 2 (TG2) is a specific autoantigen of CD, the Tissue TransGlutaminase 6 (TG6) is distributed in the central nervous system and seems to be related to neuropsychiatric disorders. Józefczuk et al. detected exclusively anti-TG6 IgA and IgG, and AGA-IgG respectively in 4 and 21 out of the 77 ASD patients evaluated. Neither anti-TG2, nor Intestinal Fatty Acid Binding Proteins (I-FABP) or zonulin, which are markers of an impaired gut barrier, were found in their sera [37]. These findings revealed an inconsistency in the hypothesis of protein translocation through the impaired intestinal epithelium to the systemic circulation and the blood-brain barrier, which would reach the brain and affect children’s behavior [37]. Not to mention that, both for TGA and anti-Deamidated Gliadin Peptide (DGP), a biochemical test seropositivity may be a transient phenomenon in young children that does not necessarily predict the development of CD, as it may be the case, for example, for subjects with HLA gene susceptibility who have ongoing infectious events [38].

## Conclusion

In conclusion, our data validate a weakness of association between autism spectrum disorder and celiac disease. On the basis of our results, a regular screening for CD in patients with ASD is not recommended and we should pay attention to an overestimation and overdiagnosis of CD, in particular in very young children with autism spectrum disorder. Nevertheless, we suggest that ASD patients who present with persistent typical and/or atypical gastrointestinal manifestations should be screened for CD, considering “pain” as a symptom that might be expressed as an atypical behavior.

## Electronic supplementary material

Below is the link to the electronic supplementary material.

Supplementary Material 1

## Data Availability

Drs. Zambrano, Morabito, Romaniello and Mani had full access to all data in the study and takes responsibility for their integrity as well as accuracy of data analysis. The main data generated and/or analyzed are also included in this published article. The overall datasets generated and/or analyzed are not publicly available in order to protect the children’s and their families’ privacy, but are available from the aforementioned authors on reasonable request.

## Abbreviations

M: Male
F: Female
ESPGHAN: European Society of Paediatric Gastroenterology, Hepatology and Nutrition
p or P or P-value: Probability value
OR: Odds Ratio
ASD: Autism Spectrum Disorder
CDC: Centers for Disease Control and Prevention
ADDM: Autism and Developmental Disabilities Monitoring
DSM: Diagnostic and Statistical Manual of Mental Disorders
ADHD: Attention-Deficit/Hyperactivity Disorder
CI: Confidence Interval
CD: Celiac Disease
HLA: Human Leukocyte Antigen
CI: Confidence Interval
TGA or TTG or tTG: Anti-Transglutaminase Antibodies
AGA: Anti-Gliadin Antibodies
EMA: Anti-Endomysium Antibodies
CNS: Central Nervous System
HZ: Hazard Ratio
Ig: Immunoglobulin
IFI: Indirect Immunofluorescence Technique
EGDS: Esophagus-Gastro-DuodenoScopy
PCR-SSP: Polymerase Chain Reaction to Sequence Specific Primers
ADI-R: Autism Diagnostic Interview-Revised
ADOS-2: Autism Diagnostic Observation Schedule-Second Edition
GFD: Gluten-Free Diet
No.: number/Id of patient
n.p.: not performed
n.a.: not available
etc.: et cetera
TG2: Tissue TransGlutaminase 2
TG6: Tissue TransGlutaminase 6
I-FABP: Intestinal Fatty Acid Binding Proteins
DGP: Anti-Deamidated Gliadin Peptide
