# Supplementary material for: Celiac disease in autism spectrum disorder: data from an Italian child cohort
Source: Ital J Pediatr. 2023 Jul 3;49:79. doi: 10.1186/s13052-023-01484-x (PMC10318650; doi:10.1186/s13052-023-01484-x)
Supplement: Supplementary file 1 — Supplementary Material 1 [file 13052_2023_1484_MOESM1_ESM.docx]

Bosisio Parini (LC), April - May 2023

Dear Editor,

We are grateful to the editors and reviewers for their time and constructive comments on our manuscript.

We have implemented their comments and suggestions and wish to submit a revised version of the manuscript for publication in the IJP.

**REVIEWER'S REPORTS**

**Reviewer #1**

Comments to the Author

The article address a controversial problem: the co-occurrence of celiac disease in patients with autism spectrum disorder and its role in the etiopathogenesis of ASD. The authors studied the seroprevalence and the prevalence of CD in a cohort of patients with ASD in comparison with a cohort of healthy children.

1. *The authors mention that they counted the subjects screened less seropositive individuals (in total 188 patients); it is not clear what means less seropositive and if these 188 patients are out of 196 screened subjects.*

Thanks very much for this observation. The number 188 represents the CD seronegative subjects: 196 screened ASD subjects – 8 CD seropositive subjects (cases) = 188 CD seronegative subjects (controls).

We have amended the relevant sentence in the text to clarify this point (sentences n. 178-179). We have also added a clarification in the legend of Table 2.

2. *In the table 2, the number of controls for seroprevalence is 188, although in text is mentioned that CD seroprevalence was established in 8 out of 196 screened subjects; also, it is not clear what represents the number 218 of controls for prevalence, a number which was not mentioned in the text.*

Thanks very much for this comment. The number 218 represents the patients in whom the diagnosis of full-blown CD has been excluded (controls) among the 223 eligible ASD patients. In particular: 223 eligible ASD subjects of our cohort – 5 ASD subjects with overt CD (CD-cases) = 218 ASD subjects without overt CD (controls).

We would also like to clarify that the methodologies and statistical calculations were performed by replicating the methods of the study by Gatti et al. (2020) [15] – that we described in sentences from n. 146 to 153 – in order to make the two cohorts (ours and Gatti’s) comparable and to minimize the statistical biases.

We have added a clarification of the aforementioned points in the legend of Table 2.

3. *There is a difference regarding the age interval between the two cohorts: 1.6 - 12.8 years for ASD cohort and 5.0 - 11.0 years for healthy
   children cohort; the authors should explain that this difference did not influence the statistical analysis.*

Thanks very much for this important observation and for the suggestion. To conduct our study, we performed a thorough literature review looking for a cohort of healthy preschool-school children in whom the prevalence of CD seropositivity and overt CD were assessed according to the ESPGHAN guidelines in order to make the two cohorts comparable and to minimize the statistical biases. To the best of our knowledge, Gatti's [15] cohort of healthy children aged 5.0 - 11.0 years is one of the largest (and therefore statistically reliable) and closest in age to that of the children in our sample. We have not found any literature studies focused (only) on healthy preschool-aged children, for whom we are not even sure that a mass screening would not lead to ethical problems. Furthermore, in support of the comparability between our ASD cohort and that of Gatti’s, in the text we have cited the study by Prosperi et al. (2021) [27], methodologically similar to ours (sentences from n. 107 to 109): *"A recent cross-sectional retrospective study conducted on a large Italian cohort of school-age children with ASD (average 7.2 years) ruled out a significant difference in the prevalence of CD compared to the general Italian population (respectively 2.18% vs. 1.58, P-value (P)= 0.36) [27]"*. In particular, in Prosperi’s ASD children sample (average age 7.2 years) a prevalence of 2.18% of subjects with full-blown celiac disease was found, a result comparable to that obtained by us (2.24%) in a cohort of ASD children with a mean age of 4.3 years and a median age of 3.6 years. Finally, some studies that we have already cited in the text (sentences from n. 237 to 253) [35, 37, 38], discuss the evidence of an increased immune reactivity and sensitivity to gluten in individuals with ASD, even in the absence of overt CD, which would be more evident in younger children rather than older ones.

Supported by literature data, we therefore believe that the early age of ASD subjects of our cohort influenced minimally the results of our study and that the potential selection bias (of epidemiological nature) could possibly be interpreted as a risk in the opposite direction due to the immaturity of the immune system and to the susceptibility to infections in genetically predisposed preschool children [38] (sentences from n. 250 to 253), i.e. we may have obtained a higher CD seropositivity rate than we could have obtained in older children.

**Reviewer #2**

Comments to the Author

This paper describes the association between two relative common disorders, ASD and CD, providing some suggestion regarding the need for specific screening of CD in ADS patients.

It is an overall simple but well designed study.

1. *My main criticism concerns the selection of cases in this case-cpntrol study. In a case control study groups of patients must be comparable for the main relevant epidemiological features. For example, since the mean age of your ASD group is 3.6 whereas it is 5 in the control group, it must be stated somewhere in the results that the two groups of patients are comparable (if they are!) for ages. Otherwise a comparison of prevalences is surely biased.*

Thanks very much for this important observation, that is an issue also raised by the Reviewer 1 (answer n. 3). To conduct our study, we performed a thorough literature review looking for a cohort of healthy preschool-school children in whom the prevalence of CD seropositivity and overt CD were assessed according to the ESPGHAN guidelines in order to make the two cohorts comparable and to minimize the statistical biases. To the best of our knowledge, Gatti's [15] cohort of healthy children aged 5.0 - 11.0 years is one of the largest (and therefore statistically reliable) and closest in age to that of the children in our sample. We have not found any literature studies focused (only) on healthy preschool-aged children, for whom we are not even sure that a mass screening would not lead to ethical problems. Furthermore, in support of the comparability between our ASD cohort and that of Gatti’s, in the text we have cited the study by Prosperi et al. (2021) [27], methodologically similar to ours (sentences from n. 107 to 109): *"A recent cross-sectional retrospective study conducted on a large Italian cohort of school-age children with ASD (average 7.2 years) ruled out a significant difference in the prevalence of CD compared to the general Italian population (respectively 2.18% vs. 1.58, P-value (P)= 0.36) [27]"*. In particular, in Prosperi’s ASD children sample (average age 7.2 years) a prevalence of 2.18% of subjects with full-blown celiac disease was found, a result comparable to that obtained by us (2.24%) in a cohort of ASD children with a mean age of 4.3 years and a median age of 3.6 years. Finally, some studies that we have already cited in the text (sentences from n. 237 to 253) [35, 37, 38], discuss the evidence of an increased immune reactivity and sensitivity to gluten in individuals with ASD, even in the absence of overt CD, which would be more evident in younger children rather than older ones.

Supported by literature data, we therefore believe that the early age of ASD subjects of our cohort influenced minimally the results of our study and that the potential selection bias (of epidemiological nature) could possibly be interpreted as a risk in the opposite direction due to the immaturity of the immune system and to the susceptibility to infections in genetically predisposed preschool children [38] (sentences from n. 250 to 253), i.e. we may have obtained a higher CD seropositivity rate than we could have obtained in older children.

2. *I also suggest to change in the conclusion the expression "is not strongly recommended" in is not "recommended on the basis of our results" because the evaluation of the degree of reccomandation is scientifically performed in other kind of papers such as position papers or guidelines.*

Thanks for this important suggestion. We have changed the sentences n. 257-258 according to the indication.

**EDITOR'S REPORTS**

**Editor #1**

Comments to the Author

Before we can further process it you are kindly requested to make the following corrections to meet the journal's requirements (please also refer to the Submission Guidelines):

1. *Mismatch Author Names*

*We notice that there are discrepancies in the author names given in the manuscript and the author names in the submission system (Barbara Parma, Valeria Morabito, Silvia Borini, Roberta Romaniello, Massimo Molteni, Elisa Mani, Angelo Selicorni). Please ensure it appears exactly the same.*

Thanks for this important observation. We are sorry, but we didn’t find any discrepancies in the author names given in the manuscript and the author names in the submission system. Attached are the screenshot of the submission system pages and the revised paper. Please, if the problem persists let us know.

2. *Missing the "Consent for publication", please provide.*

*If your manuscript contains any individual person’s data in any form (including any individual details, images, or videos), authors should seek written and signed consent to publish the information from the patients or their guardians prior to submission. If the person has died, consent for publication must be obtained from their next of kin. The submitted manuscript must include a statement to this effect in the 'Consent for publication' section, as follows: "Written informed consent for publication was obtained.".*

*If your manuscript does not contain data from any individual person, please state “Not applicable” in this section.*

Thanks for this important suggestion. According to the indication, we have added a sentence on informed consent in the text (sentences n. 133-134) and in the “Declarations” section (sentences n. 289-290).

**Editor #2**

Comments to the Author

Before we can further process it you are kindly requested to make the following corrections to meet the journal's requirements (please also refer to the Submission Guidelines):

1. *Mismatch Author Names*

*We notice that there are discrepancies in the author names given in the manuscript and the author names in the submission system (Barbara Parma, Valeria Morabito, Silvia Borini, Roberta Romaniello, Massimo Molteni, Elisa Mani, Angelo Selicorni). Please copy the names in the submission system and paste them to your manuscript file and ensure it appears exactly the same.*

Thanks for this important observation. We have changed the author names in the manuscript as indicated. Please, if the problem persists let us know.

2. *Under the "Ethics approval and consent to participate", please provide the correct statement below.*

*- A statement to confirm that all experimental protocols were approved by the name of the ethics committee and the committee’s reference number if appropriate. If a study has been granted an exemption from requiring ethics approval, this should also be detailed in the manuscript (including the name of the ethics committee that granted the exemption).*

*- A statement to confirm that all methods were performed in accordance with the ethical standards as laid down in the Declaration of Helsinki and its later amendments or comparable ethical standards.*

*- A sentence confirming that written informed consent was obtained from all participants or if participants are under 16, from a parent and/or legal guardian. Include a statement on ethics approval and consent (even where the need for approval was waived)*

Thanks for this important suggestion. We have changed the section "Ethics approval and consent to participate" as indicated.

3. *Please provide the "Consent for publication" in separate headings. It should not be included in the "Ethics approval and consent to participate".*

*Consent for publication*

*If your manuscript contains any individual person’s data in any form (including any individual details, images, or videos), authors should seek written and signed consent to publish the information from the patients or their guardians prior to submission. If the person has died, consent for publication must be obtained from their next of kin. The submitted manuscript must include a statement to this effect in the 'Consent for publication' section, as follows: "Written informed consent for publication was obtained.*

Thanks for this important suggestion. We have created the two sections "Ethics approval and consent to participate" and “Consent for publication” as indicated.

**Editor #3**

Comments to the Author

Before we can further process it you are kindly requested to make the following corrections to meet the journal's requirements (please also refer to the Submission Guidelines):

1. *Please upload one manuscript file only in your submission. If you wish to indicate changes made, please upload a separate version labeled "Manuscript with marked changes". Please upload this as a “Supplementary Material” file.*

Thanks for this suggestion. We proceeded as you requested.

2. *Mismatch Author Names*

*We notice that there are discrepancies in the author names given in the manuscript and the author names in the submission system (Barbara Parma, Valeria Morabito, Silvia Borini, Roberta Romaniello, Massimo Molteni, Elisa Mani, Angelo Selicorni). Please copy the names in the submission system and paste them to your manuscript file and ensure it appears exactly the same.*

Thanks for this important observation. We have changed the author names in the manuscript and in the submission system.

3. *Under the "Ethics approval and consent to participate", please provide the correct statement below.*

*- A sentence confirming that written informed consent was obtained from all participants or if participants are under 16, from a parent and/or legal guardian. Include a statement on ethics approval and consent (even where the need for approval was waived).*

Thanks for this important suggestion. We have changed the section "Ethics approval and consent to participate" as indicated.

4. *Please provide the "Consent for publication" in separate headings. It should not be included in the "Ethics approval and consent to participate".*

*Consent for publication*

*If your manuscript contains any individual person’s data in any form (including any individual details, images, or videos), authors should seek written and signed consent to publish the information from the patients or their guardians prior to submission. If the person has died, consent for publication must be obtained from their next of kin. The submitted manuscript must include a statement to this effect in the 'Consent for publication' section, as follows: "Written informed consent for publication was obtained.".*

*If your manuscript does not contain data from any individual person, please state “Not applicable” in this section.*

Thanks for this important suggestion. We have created the two sections "Ethics approval and consent to participate" and “Consent for publication” as indicated.

5. *Please remove the duplicate file in the file inventory and retain the updated file.*

Thanks for this suggestion. We proceeded as you requested.

Kind Regards,

Dr. Stefania Zambrano et al.
